# Enhancing Collaborative Ideation in Organizations

**DOI:** 10.3389/fpsyg.2018.02024

**Published:** 2018-10-22

**Authors:** Paul B. Paulus, Jonali Baruah, Jared B. Kenworthy

**Affiliations:** ^1^Department of Psychology, University of Texas at Arlington, Arlington, TX, United States; ^2^Department of Psychological Sciences, Tarleton State University, Stephenville, TX, United States

**Keywords:** group creativity, collaborative ideation, organizational innovation, team innovation, brainstorming, meetings

## Abstract

Extensive research and theory has focused on organizational innovation and the organizational factors that influence that innovation. Research on teams has highlighted a similar set of factors as important for team innovation. However, these literatures have not provided a clear picture of the key factors that influence the collaborative idea exchange processes that occur in teams and organizations. The literature on collaborative ideation has provides a useful theoretical and empirical basis for understanding these processes and the conditions required for optimizing creativity in group interactions. We provide the theoretical and empirical basis for a pragmatic approach to enhancing collaborative innovation processes in various organizational settings and highlight additional research needs and future directions.

## Introduction

There is a considerable literature on team and organizational innovation. A recent special protectissue of the American Psychologist provided detailed reviews of the teams literature including team innovation (Thayer et al., 2018). Team innovation is influenced by many factors including the composition of the team, its diversity, the team experience, the team context or climate and leadership (Paulus et al., 2012; Reiter-Palmon, 2018).

Organizational innovation involves a similar series of factors such as leadership, support, and psychological safety (Crossan and Apaydin, 2010; Anderson et al., 2014). Key factors in both team and organizational innovation are the interactional processes. Factors that influence the innovation process include the degree and quality of communication, degree of conflict, social networks, social distance, psychological safety, and means of communication (Hülsheger et al., 2009; Farh et al., 2010; Paulus et al., 2012; Edmondson and Lei, 2014). Although there has been significant progress in this area, some key limitations remain. For example, many researchers rely primarily on self-reports of innovation which may not accurately reflect actual performance (Paulus et al., 1993; Reiter-Palmon et al., 2012). Also, there is a lack of theoretical integration of the individual, team, and organizational factors (Anderson et al., 2014).

Although there is now considerable consensus about the important factors in organizational and team innovation, most of these factors are beyond the control of individual members of the team and organization. Most of the suggestions for application relate to specific procedures or approaches that are directed by organizational leaders (Thayer et al., 2018). These individuals are typically not well-prepared for such roles. Few have exposure to the relevant literatures, and they may rely on consultants or “gurus” to provide simplistic suggestions for enhancing innovation, such as de Bono’s (1985) Six Hat approach for which there is no published evidence (Dingli, 2009), or simplistic approaches to brainstorming (Anderson et al., 2014). Furthermore, although companies typically endorse the importance of innovation, few know how to do it (Almquist et al., 2013) or may not be supportive of the types of people and processes required for generating truly creative ideas. Moreover, there is a strong bias to feasible ideas, which may limit the development of more novel ideas (Baer, 2012; Mueller et al., 2012).

In contrast to the literature on organizational and team innovation, there is a literature on the group creativity process that often involves ad-hoc groups in controlled settings (Paulus and Coskun, 2013; Paulus and Nijstad, in press). This research includes experimental interventions and precise assessment of theoretical models. This type of research is difficult to accomplish in organizations. Although companies tend to be supportive of survey studies, very few companies are willing to accommodate the intrusions into the work environment required to assess the impact of various group process interventions on the organization’s innovative outcomes. Thus intervention studies on organizational innovation are rare (Anderson et al., 2014). The processes involved in group creativity studies are the same as those involved in real world settings and many of the results align nicely with those of the team literature (Paulus and van der Zee, 2004; Paulus et al., 2012). Thus it is ironic that this literature is often ignored in reviews of team innovation (e.g., Hülsheger et al., 2009; van Knippenberg, 2017), presumably in part because of its predominant focus on the idea generation or brainstorming process, and because these do not involve “real people” in real world contexts. Others suggest that the brainstorming process is not an effective means of enhancing organizational innovation, based on the experience of organizational development consultants (Skilton and Dooley, 2010; Basadur et al., 2012). However, that reflects a misunderstanding of the focus and contributions of the scientific literature on collaborative idea generation. This literature is very broad and diverse in its focus (Paulus and Nijstad, in press) and has a strong theoretical underpinning. Because many of the studies are done under controlled conditions, these studies have also facilitated the development of theoretical models that have implications for team processes in organizational contexts (Nijstad et al., 2003; Paulus and Brown, 2003, 2007; Nijstad and Stroebe, 2006; De Dreu et al., 2011; Paulus et al., 2012). Furthermore, the literature on organizational innovation primarily focuses on individual-level rather than team-level effects (Anderson et al., 2014). Although the various factors highlighted in the research on team and organizational innovation are undoubtedly important, their impact will be much reduced if the interaction processes in the organization are not optimal for the creative process.

In sum, one of the key gaps in the literature is the link between the research and theory on collaborative creativity and organizational innovation. Thus, the focus of this paper is on the effective utilization of groups and teams in the creative idea generation processes of organizations. We summarize some of the key findings of the literature on collaborative ideation and suggest applications to organizational contexts. Much of this research has been done from a brainstorming perspective (see Paulus and Kenworthy, in press). The brainstorming term was popularized by Osborn (1957) for a procedure in which groups focused on generating as many ideas as possible in a short period of time without concern for quality. Many studies on ideational creativity in groups have continued to use this type of approach to enlighten the key processes and factors related to enhanced creativity in idea sharing groups. This research and the related theoretical developments have implications for any context in which information and ideas are shared with a focus on solving a problem or developing innovative new directions or products. Our aim is to suggest a practical approach to the different aspects the collaborative ideation process in organizations by providing specific suggestions for best practices that are under the control of the participants and/or the group or team leaders.

We will outline the theoretical basis for collaborative ideation and discuss the implications of the theory and empirical literature for its conduct in the various phases of the innovation process. Initially groups need to be appropriately prepared for the ideation session and their composition needs to be decided. The collaborative ideation process can be conducted by the use of different modalities such as writing or talking. The group sessions can be broken up by brief breaks or sessions for individual reflection. When the ideation sessions are complete, the ideas need to be evaluated and elaborated or refined for potential use. We also discuss the broader context for the best practices. The practices that are critical for effective ideational collaboration and evaluation can be applied to problem solving meetings. The degree of organizational support for the use of optimal processes is critical for ensuring effective ideational collaboration in structured group sessions or meetings. We conclude by highlighting some of the best practice gaps in the literature and the related future directions.

## Theoretical Background

Generating ideas in groups obviously involves the process of sharing ideas among group members. It is presumed that such sharing process will stimulate the generation of ideas that might not otherwise have occurred because of the mutual cognitive stimulation that results from idea sharing (Nijstad et al., 2003; Paulus and Brown, 2003, 2007; Nijstad and Stroebe, 2006). These ideas can result in conceptual combinations as group members build on each other’s ideas (Kohn et al., 2011). The elaboration processes involved in this building on others’ ideas are key factors in enhancing the novelty of ideas in groups with diverse perspectives (Coursey et al., in press b; van Knippenberg et al., 2004). Such collaborative ideation processes require that group members carefully balance the search process of their own knowledge base with attention to the ideas shared by other group members (Brown and Paulus, 2002).

Group members also need to be highly motivated for creative collaboration. This motivation can derive from personal (e.g., intrinsic motivation, Amabile, 1996) or external sources (e.g., rewards; De Dreu et al., 2011). However, the larger the group, the less motivated group members may be to contribute. They may feel that their efforts are fairly dispensable given the large group size (Kerr and Bruun, 1983) or they may feel less motivated to contribute (*social loafing*; Karau and Williams, 1993). Group members may also be influenced by the low performance of other group members and may move their performance downward unless there is a strong incentive not to (*downward social comparison*; Paulus and Dzindolet, 1993). Providing explicit challenging goals or some degree of competition among group members can help overcome the potential of motivation losses in groups (Paulus and Dzindolet, 1993, 2008; Larey and Paulus, 1995; Roy et al., 1996).

Although the theoretical perspectives of collaborative ideation suggest positive outcomes for creativity, often the interaction processes in groups may not be well-designed for the effective sharing of ideas and building on them. In face-to-face group settings only one person can effectively share ideas at one time, limiting the opportunity to share ideas in a limited time frame, a factor called *production blocking* (Diehl and Stroebe, 1987). While one is waiting to present one’s ideas, it may be difficult to hold these ideas in mind while also carefully attending to and building on the shared ideas. It is often the case that the primary sharing is done by only a subset of group members (Paulus and Dzindolet, 1993). Moreover, in groups there may be an implicit pressure for consensus or agreement (Janis, 1972; Paulus, 1998), and thus group members may quickly focus on only a limited range of superficial ideas (Larey and Paulus, 1999) and tend not to persist in order to fully tap the group’s potential.

## Gathering and Preparation; Getting Groups Ready for Effective Collaborative Ideation

Groups can be formed in different ways. Some groups may already exist as a unit or a team. Other groups may be explicitly formed to represent different areas of expertise, experience, or interest. Another possibility is to allow groups to self-select for a creativity session. This might increase motivation for the task or allow people who work well-together or enjoy interacting to be in the same group. Research has not been done on the impact on self-selection. However, the research on the importance of autonomy in individual creativity suggests this might be a useful approach in some cases (e.g., Amabile, 1996). In whatever fashion groups are formed, it would be helpful for them to have some time to become familiar and develop some degree of cohesion (Uzzi and Spiro, 2005; Fleming et al., 2007). This may make it more likely that group members will feel comfortable working each other on the various phases of the group creative process.

When groups gather for a creativity session it is important to provide the appropriate structure and instructions. Most formal brainstorming sessions use the Osborn rules of focusing on quantity, saying whatever comes to mind, not criticizing shared ideas, and building on the shared ideas (Osborn, 1957). There is some evidence for the utility of these rules, with the best evidence for the importance of focusing on quantity initially rather than quality (Paulus et al., 2011). The quantity rule seems to facilitate a semantic flow of ideas since any idea that comes to mind can be shared without concern for fit or quality. Focusing immediately on quality will tend to inhibit sharing of ideas because it creates an evaluative atmosphere, which tends to limit uninhibited idea generation (*evaluation apprehension*; Diehl and Stroebe, 1987; Camacho and Paulus, 1995). One additional rule which can be very helpful is to suggest that group members keep their suggestions fairly short by not elaborating or telling stories. Such story-telling is very common in most groups and limits the number of ideas that can be generated in a limited time period. Adding such an instruction can greatly increase the number of ideas generated in groups (Putman and Paulus, 2009). Although these rules are typically used in formal brainstorming sessions, the basic principles that underlie these rules are relevant for any type of collaborative idea exchange process or meeting. Participants should feel safe to share their ideas openly without concern for negative feedback. Encouraging the exchange of a large number of ideas will increase the probability of generating highly novel ideas. Encouraging group members to present their ideas in an efficient manner will increase the ability of the group to generate a good number of ideas in a limited time period.

The idea generation topics can of course vary greatly in their complexity and breadth. A company might want to have groups think broadly about ways of improving the overall functioning of the organization or simply find a better way to market a product. However, no matter the characteristics of the problem, it is beneficial to have group members discuss different ways to frame a problem prior to the idea generation stage (Reiter-Palmon and Robinson, 2009). It also appears to be useful to break the problem into subcomponents (Dennis et al., 1996; Coskun et al., 2000; Deuja et al., 2014). This allows for a deeper search within each of the subcomponents or categories of the problem and for the generation of more novel ideas (Baruah and Paulus, 2011; Rietzschel et al., 2014). The typical cognitive search process involves tapping the most obvious or common ideas first; only later will the more rare and novel ideas surface (Paulus and Brown, 2003; Nijstad and Stroebe, 2006; Baruah and Paulus, 2016; Puccio et al., 2018). When the problem to be discussed is presented in its broad form, it is likely that there will not be a deep search process of all the different elements of the problem. However, if the problem is presented in various subcomponents, each of the subcomponents will get at least some attention and the potential for deeper exploration.

It is important for groups to have some training and experience with effective group idea generation. Doing it efficiently is not natural. In order to enhance both the quantity and quality of the ideas generated, it is important to conduct a number of sessions in which participants practice using the guidelines for effective idea exchange, being attentive to one another, taking turns, and building on each other’s ideas, and are provided feedback about their performance on these behaviors (Baruah and Paulus, 2008). Similarly, it may be useful to have some facilitators monitor the process and encourage appropriate behaviors and broad involvement for inexperienced groups (Oxley et al., 1996; Kramer et al., 2001).

## Composing Groups to Enhance Collaborative Creativity

People vary considerably in their feelings about working in groups and teams. Research suggests that those who like working in groups or teams will generally perform better in these settings (Larey and Paulus, 1999; Aguado et al., 2014). Similarly, those with a strong sense of competence or efficacy about a particular task are more likely to enjoy working on such a task in a group setting than those who do not feel so confident since the group setting allows them to demonstrate this competence (Taggar, in press). In contrast, those who are generally uncomfortable in group settings or have social interaction anxiety are not likely to thrive in group settings (Camacho and Paulus, 1995). Thus as a general policy it might make sense to allow individuals to self-select their involvement in group projects. However, if individuals who prefer group contacts are generally agreeable, this might not be beneficial if the group context requires an honest exchange of perspectives and discussion of conflicting perspectives is needed (Nemeth and O’Conner, in press; Stasser and Abele, in press). Thus group composition may need to be adjusted depending on the task. In general, for collaborative creativity it may be useful if the participants are relatively high on openness to experience, conscientiousness, and extraversion (Bell, 2007; Feist, 2010; Coursey et al., in press a) and have a diverse range of experiences including exposure to other cultures (Tadmor et al., 2012).

Although there is considerable evidence that certain individual characteristics are related to increased creativity in groups and teams, it may be difficult to utilize this information in work settings. Prior to employment, employees might be tested for these characteristics to use them as input for the hiring process. Hiring people who are high in conscientiousness and low on neuroticism is certainly a good strategy. However, it is not so clear that one would want to select based on introversion/extraversion or openness to experience. In work settings a wide range of tasks and settings may differentially require introverted or extraverted interaction styles. Those who are able to function effectively in social contexts but are also able to focus effectively on solitary tasks may be ideal for most work settings. Openness to experience may be beneficial for creativity thinking but not all aspects of the work environment. It may also be important to have a diversity of personality types in an organization so that employees can self-select to the aspect of the work environment that is most suited for them. In addition, groups with a diverse personality composition may be better suited for a range of tasks. For example, for creativity tasks the group members who are high on openness to experience and extraversion may be particularly useful in the divergent idea generation stage. However, those who are high in conscientiousness and introversion may be more effective in the development and implementation stage of innovation that requires a high degree of reflection, persistence, and the resolution of conflicting perspectives (Cullen-Lester et al., 2016). One could also allow group members to change groups at different points in the process since that can provide some additional stimulus for continued ideation in the group (Choi and Thompson, 2005; Nemeth and Ormiston, 2007; Levine and Choi, in press).

If the task requires diverse expertise, personality types or perspectives, the group should obviously include that type of diversity. However, to minimize the problems related to large groups, it would be best to keep the group as small as possible while accommodating the required diversity (Cummings et al., 2013). Research on diversity and creativity has found that both demographic and expertise diversity can enhance creativity (van Dijk et al., 2012; Paulus et al., in press). The van Knippenberg et al. (2004) categorization-elaboration model posits that diversity in teams can be associated with low cohesion and relationship conflict, but the presence of diverse knowledge and perspectives may motivate cognitive elaboration of task related information, thus benefiting team creativity. However, conceptual gaps can inhibit the creative process in cognitively diverse groups (Cronin and Weingart, 2007) since there will be difficulties in understanding each other’s ideas and building on them. Experience as a group or team can enhance mutual understanding and thus enhance team innovation (Cummings and Kiesler, 2008). Demographic diversity can also inhibit creativity if group members are somewhat uncomfortable and feel constrained in the free expression of ideas because of concern about potential differential sensitivities (Bell et al., 2011). In terms of cultural sensitivities, the literature reveals that cultural diversity can have a negative impact on interpersonal relationships (e.g., development of mistrust due to cultural differences), but it benefits team creativity by yielding unique ideas and perspectives (Shin and Zhou, 2007). Similarly, Stahl et al. (2010) reported that cultural diversity was associated with low cohesion, trust, morale, and attraction but with a high level of team creativity.

Experience as a group or team and the development of some degree of familiarity and trust among the group members may increase the comfort level and feelings of psychological safety in such diverse groups (Edmondson and Lei, 2014). Group members who have a positive attitude to diversity or are primed to have a positive expectation of diversity (Homan et al., 2007; Nakui et al., 2011; van Knippenberg et al., 2013) tend to benefit most from diversity. Thus programs to encourage positive interactions and perspectives related to diversity will likely enhance the creative potential of diverse group interactions.

## Structuring the Collaborative Ideation Process

### The Use of Different Modalities

When people think of brainstorming they generally think of a group of people exchanging ideas by talking. That is indeed the most commonly used method. Participants tend enjoy it and perceive it as effective (Paulus et al., 1993). Verbal idea sharing in groups is better than not sharing ideas at all since a good number of ideas will be generated. However, as suggested earlier, there are a number of factors which limit its effectiveness such as production blocking, evaluation apprehension, and downward social comparison. Thus groups of four tend to generate only about half as many ideas as the same number of individuals brainstorming alone (i.e., *nominal* groups) (Diehl and Stroebe, 1987; Mullen et al., 1991). Verbal idea sharing also requires some way of recording the ideas. Some or all group members may be tasked with keeping track of the ideas on sheets of paper for later evaluation. This becomes an additional factor in slowing down the rate of idea generation and potentially reducing attention to the shared ideas.

A highly recommended alternative is to use an electronic method for exchanging ideas (Dennis et al., in press; Nunamaker et al., 1987; DeRosa et al., 2007). There are many platforms that enable such a process and which allow participants to generate ideas as they occur and to examine the shared ideas at will. These systems also allow for voting on the best ideas at some point and collectively deciding on the best ideas. This method tends to be more efficient in generating more ideas than the verbal method. Although the electronic method appears useful for the generation of ideas, its utility for evaluation appears to be in doubt. Evaluation is better accomplished in a face-to-face setting (Dennis, 1996; Kerr and Murthy, 2004). Furthermore, participants are typically free to ignore each other’s contributions and focus only on generating their own. So this method may not effectively tap the collective creativity of the group.

Even though electronic idea exchange appears to be a useful way of sharing ideas in groups, in organizational contexts its utility may be limited, especially in co-located groups. The use of such systems requires a significant investment and often the use of experts or facilitators to manage the process. Another approach may provide more flexibility as well as more creative potential—sharing ideas by writing (Heslin, 2009). This can involve sharing ideas on slips of paper or post-it notes. Research on paradigms that involve passing of slips of papers among group members suggest that it may be a quite effective approach (Paulus and Yang, 2000; Goldenberg et al., 2013; Litcanu et al., 2015). Using a writing procedure in groups enhanced idea generation in a high technology company (Paulus et al., 2015), and in a recent workshop we found that this procedure yielded twice as many ideas as the verbal approach in a short session (Baruah and Paulus, 2018). Typically, a small group sits at table and each member is provided with a set of slips. Once someone writes their idea on a slip, it can be put in a pile in the middle of a table (pooling) so that other group members can read the ideas when they wish (Michinov, 2012). Another alternative is to pass the slips from one person to another for reading. When it comes back to the originator it can be placed in a pile in the center of the table (Korde and Paulus, 2017). The advantage of the latter approach is that it requires attention to the shared ideas. However, the forced reading may interrupt the personal ideation process and take time away from individual brainstorming. Another variation involves participants writing ideas on the slips that come to them as a way to encourage building on ideas (Paulus and Yang, 2000). Thus they have the option of writing on a received slip or simply passing it on and generating a new slip. There have been no comparisons of the different ways of sharing slips or of the relative effectiveness of the “post-it notes” approach. Thus, although a number of studies have demonstrated that writing groups can exceed the performance of nominal groups (Paulus and Yang, 2000; Paulus et al., 2015), we do not know which approach is best.

The writing procedure has a number of advantages. It is simple to conduct, requires minimal training, ensures a fairly equal involvement in the sharing process, and enhances attention to and elaboration on shared ideas. It limits production blocking, reduces evaluation apprehension since it minimizes awareness of the sources of the ideas, and enhances motivation to generate ideas since all members are typically very active in sharing ideas and participants may feel the pressure to generate their “fair share.” So, from a theoretical perspective, this is an ideal paradigm. However, it is not a familiar procedure to most people. Thus it requires some experience and demonstration of its benefits to encourage its adoption. It is possible that using a “hybrid” approach that allows group members to make brief comments during the writing process will increase the extent to which participants find this process engaging.

The right group size for the different modalities is an interesting issue. When we ask audiences at talks for an ideal size for group ideation, the answer is typically greater than four. In regard to verbal idea generation groups, however, the larger the group, the fewer ideas per person (Bouchard and Hare, 1970). That is not surprising since the more people in the group, the less opportunity each individual has to contribute. The ideal group in terms of ideas per person is a pair (Mullen et al., 1991). However, if a broader range of expertise is required, the general recommendation is keeping the group as small as possible while accommodating the needed diversity. Group size seems to be less of a problem with electronic idea generation, where groups of eight or more tend to produce more ideas than smaller groups (DeRosa et al., 2007). Thus larger groups appear to benefit from the exposure to a large number of ideas in that paradigm without experiencing the blocking that would occur in large verbal groups. There are no studies examining group size with the writing method. The benefits of collaborative writing might diminish as group size increases because larger groups would require investing more time in reading rather than in generating. Smaller groups may provide a more appropriate balance of sharing and reading.

### Alternating Group and Alone Sessions

We have discussed the pros and cons of different methods of sharing ideas in relation to one another and to their nominal group comparisons. One important measure of group effectiveness is the extent to which group ideation leads to better outcomes than individual ideation. However, in real-world contexts the idea generation process tends to involve both solitary and collaborative ideation. It is likely that some balance of individual and group ideation is ideal. Before joining a group discussion it may be useful to think ahead of time about the problem and to begin generating one’s own ideas. This ensures that the group members can begin the group session with a solid flow of ideas which can in turn stimulate additional ideas. However, no matter what paradigm is used, at some point it may be useful to reflect individually on the shared ideas and to build on them. Many of the associations generated may not be tapped during the group session and may decay over time. It may be ideal to provide some mix of alone and group idea generation. Thus far this has only been demonstrated with the writing method (Paulus and Yang, 2000; Paulus et al., 2015; Korde and Paulus, 2017), but theoretically it should also work for the other paradigms. At this point we do not know the ideal balance of alone/group time allocation. It will probably vary by task and paradigm. We have only experimented with short sessions and doing so may be difficult in practice. However, it is also useful after longer sessions since there appears to be a considerable benefit of having a solitary session after a group session to fully tap all of the cognitive stimulation generated by the group sharing process (Paulus and Yang, 2000). In a subsequent session the additional ideas generated by the private reflection of the individual group members can be shared to allow the group to build on these ideas (Kohn et al., 2011).

It appears that brief breaks in the ideation process can be beneficial for both groups and individuals. Only one published study has examined this issue (Paulus et al., 2006), and there is not much known about the specifics of time for breaks and what should occur during the breaks. The breaks may allow for cognitive rest and restoration of one’s attentional capacity (Berman et al., 2008). They may also enable one to overcome fixation on specific aspects of a problem (Smith, 2003) and allow for new associations to surface (Paulus et al., 2006). In one study we found that one long break or two shorter ones did not make a difference in the overall benefit for the number of ideas generated (Paulus et al., 2006). The breaks were beneficial for the writing procedure but not for the electronic one. The content of activities in the break may also be important. The research by Smith (2003) suggests that having activities during the break that differ from the focal task will be most beneficial. The study by Berman et al. (2008) suggests that exposure to nature elements during the break may be most rejuvenating. However, given the paucity of research on this issue, we feel strong recommendations about timing of breaks and their content cannot be made. We encourage organizations to experiment with different kinds of breaks during the ideation process to determine whether there are any obvious benefits of one type of break over another. However, we feel that long breaks may be counterproductive since it may interrupt the momentum and task focus gained during the collaborative ideation process.

## The Convergent Phase: Evaluation, Selection, and Development

The creative process is often considered a divergent thinking process. Indeed, much creativity involves “thinking outside the box” in the sense of trying come up with highly novel ideas. This typically requires the type of idea generation process we have discussed thus far with a focus on quantity rather than quality. However, in most cases it becomes necessary to sift through the ideas generated to select the best ones. This can be a rather intimidating task. Groups can generate 100s or 1000s of ideas in a relatively short time. Going through these ideas to pick the best ones will be a difficult task. In our laboratory it takes many hours for highly trained and motivated students to code the ideas for such dimensions as novelty and feasibility. In organizations, it may be difficult to use this type of approach. Alternatively, the ideas may be divided among a group of individuals for rating them along various dimensions and then selecting those that received the most favorable ratings for further discussion and elaboration.

The typical brainstorming approach is to generate as many ideas as possible without concern for quality to ensure the generation of a large number of ideas. The presumption is that one can subsequently focus on making these ideas more useful or feasible. However, some research suggests that this may not be necessary for collaborative ideation. Harvey and Kou (2013), in a qualitative study, found that teams that focused simultaneously on both generation and evaluation tend to direct collective attention to the ideas, promote retention of the novel ideas, and encourage building and elaboration of the ideas in final creative outputs. Thus it may not be necessary to be too strict about the focus only on generating and not evaluating as participants go through their session. A mixed focus may reduce the number of ideas but may possibly increase the average originality of ideas generated (see also Puccio et al., 2018).

The evaluation process typically occurs after the group has completed the idea generation process. The ideas could be evaluated by “outsiders,” but Faure (2004) found that those who evaluated their own ideas did a better job of picking the best ideas. One problem with the evaluation process is that groups tend to focus on feasible ideas rather than novel ideas in the selection process (Rietzschel et al., 2006; Putman and Paulus, 2009). This is understandable since very novel ideas may not seem very realistic or practical (Baer, 2012; Mueller et al., 2012). Thus groups and individuals tend not to pick ideas that are above average in novelty. For example, panels that evaluated research and development projects for funding tended to select proposals of moderate novelty rather than highly novel ones, especially if the panel had a high workload (Criscuolo et al., 2017). Rietzschel et al. (2006; 2010; 2017) have done considerable research on this topic, and they find that the generation of original ideas does not necessarily result in the selection of the good quality ideas because of the *feasibility bias*. Hence, we suggest that an effective evaluation process prior to implementation may be beneficial in offsetting this bias.

One problem with the process in the idea evaluation studies is that it requires individuals or group members to evaluate a large number of ideas. In fact, there is some evidence that groups that generate a lot of ideas tend to select less original ideas (Perry-Smith and Coff, 2011). If group members are allowed to vote on the shared ideas as they go along, this might overcome this problem. This would be easiest with electronic or writing methods. For example, at the end of a writing session, group members could make a mark on ideas they thought were highly novel or high in quality. Then they could focus on a set of ideas that received the most votes and select the best ideas from that set for further development. This might enable more of the novel ideas to make it to a final stage in which there should be a focus on making ideas more feasible or practical. In fact, it might be useful to break up the ideas or problems among subgroups. Each of these groups would evaluate and work in developing more feasible and practical solutions. At some point they might be asked to select their top choice or choices. These could then be shared with the other groups. This should be particularly compelling since groups are likely to come up with very different solutions. This will demonstrate the utility of having different groups focus separately on subsets of ideas to ensure that a number of different ideas of high quality are brought forward. These ideas can then be shared with decision makers or other groups in the organization (such as the marketing department) for further evaluation and development.

Typically, only a limited number of alternatives can be developed within the resources of the company. The process outlined above should greatly increase the quality of ideas brought forward for additional consideration.

Other than the studies on idea selection, research has generally not focused on the relationship between the divergent creativity phase and the convergent one in developing final products based on the prior ideation session. One might presume that a session that generates many novel ideas will lead to a more novel final product. However, that may not necessarily be the case. Given the feasibility bias mentioned earlier, it is likely that most novel ideas will get little consideration. Thus, there is a need for more research on convergent creative processes of building, elaborating, development and implementation, and their relationship to the prior divergent idea generation process (cf. Paulus et al., 2018).

## Making a Good Collaborative Creativity Process Part of the Organizational Culture

There is much talk about the importance of teamwork, collaboration and innovation in organizations. However, few in an organization will know how to effectively tap the collaborative creativity potential in the organization. We have outlined the processes and procedures that can be embedded in the team and collaborative processes of the organization. They provide a template for effective problem-solving meetings at all levels and for specific group ideation or team innovation activities. However, it takes positive experiences with these processes for them to gain some degree of acceptance in the organizational culture. As usual, the acceptance and use of best practices depend on the extent to which the organizational leadership demands excellence and best practices for creative or problem-solving activities. Only then will best practices become entrained in the organizational culture of the organization. One example of such a culture is IDEO corporation, which is a top product development company (Sutton and Hargadon, 1996). The employees utilize basic principles of brainstorming as part of their culture. A key factor is the strong endorsement of the collaborative creativity approach by the leadership. The founder of this company, Tom Kelley, has consulted with other organizations on developing a more innovative culture. However, the processes used at IDEO are not necessarily optimally aligned with the best practices suggested by the literature. Companies may not have to hire outside consultants once they fully comprehend the basic principles of the approach we have suggested and embed those as part of the organizational culture.

## Application to Meetings

Although we have focused on the use of explicit idea generation or problem-solving sessions, the principles highlighted can also be used for enhancing the utility of face-to-face meetings that focus on problem solutions. Thousands of such meetings occur every day and there are obvious concerns about the value of those meetings relative to the time invested and the cost in lost productivity (Rogelberg et al., 2012; Lehmann-Willenbrock et al., 2018). Lehmann-Willenbrock et al. (2018) have outlined key factors in successful meetings from the vantage point of the research evidence from a broad range of disciplines. The research and theory we have reviewed suggest some additional factors to consider. The number of people at meetings should be kept as small as possible while still including the expertise required. If it is important to have broader involvement, separate meetings with subgroups should be organized. Again, if similar solutions are suggested by most of the groups, there will be increased confidence in the correctness of the decision. But if there are significant discrepancies in suggested solutions, this will stimulate additional evaluations and hopefully a better and more integrated solution (Paulus, 1998).

Keeping meetings small will increase the opportunities for the participants to contribute to the discussion. Thus there will be an increased chance of tapping the broad diversity of perspectives and increasing the feeling that one’s opinions and ideas are valued. It might also be useful for group members to write down their ideas in a reflection session at the beginning of the meeting or prior to the meeting. If this is done during the meeting, they could be passed among the group for additional elaborations. This allows for an effective and efficient sharing of initial ideas, avoiding the problems of production blocking and domination of meetings by a limited number of group members. As these ideas are subsequently shared verbally, the group members can also be encouraged to keep writing their thoughts for potential sharing at some later point in the meeting. For important decisions, second chance meetings should be arranged in which ideas and concerns that arose in the minds of group members in the interim are shared and evaluated (Paulus, 1998).

It may also be useful to vary different interaction modalities for meetings. We have suggested mixing writing and verbal interactions in meetings, but these could also be complemented by electronic exchange in which group members continue discussions after the meeting. These discussions can then serve as inputs for future meetings in which these additional ideas or concerns are discussed.

## Pulling it All Together: Individuals, Groups/Teams, and Organizations

We have outlined in detail how to effectively utilize the principles of collaborative ideation to enhance the creative processes in groups, teams, and meetings. Although our focus has been on collaborative creativity, we should note that many of the factors that enhance collaborative creativity, such as task structure and framing, instructions, training, and breaks, can also enhance individual creativity (cf. Paulus and Coskun, 2013). Although the extensive literature in individual creativity can be of value in relation to organizational innovation, most innovative activities in organizations involve some degree of collaboration. Given the additional complexity of collaborative creativity, addressing only individual creativity may be of limited value to organizational innovation.

It is presumed that following best practices in the conduct of individual and group creativity sessions and meetings will increase the probability that the ideas being generated and evaluated are indeed a good reflection of the creative potential of the employees and the probability of development of radical or “breakthrough ideas” (Naranjo-Valencia et al., 2011; Harvey, 2014). However, we realize that many organizational factors can inhibit the extent to which such ideas are further developed and eventually implemented, such as lack of support for innovation or the lack of a system of moving ideas through the various phases of development (Anderson et al., 2014). There is also a need to balance the needs of the many routine tasks that need to be accomplished. Thus a constant focus on radical innovation may be quite disruptive (Anderson et al., 2014; Mariano and Casey, 2015). However, there should always be openness to incremental innovations that involve improvement in the organizational functioning.

Creating an effective and balanced innovation process in an organization requires sophisticated and supportive leadership from the top management team (Bledow et al., 2009; Wunderlich et al., 2014; Carmeli and Paulus, 2015). Researchers have emphasized the need for ambidextrous leadership approach in which leaders enact different styles for different phases of the innovation process. Thus a transformational or inspirational leadership style may be required to promote the early ideational phases, but the more detailed development, refinement and implementation phase may require more directive or transactional style (Rosing et al., 2011). Some have suggested an even more complex series of phases and related actions (Cropley and Cropley, 2012). This would require highly sophisticated leaders unless there is some sort of artificial intelligence support system. Until that is developed, it is likely that developing some basic degree of sophistication in leaders about how to manage and facilitate the creative processes in organizations will have to suffice (Williams and Foti, 2011; Shanker et al., 2017).

In sum, what is the recipe for a feasible approach to effectively integrating the individual, group, team and organizational innovation processes? As suggested by Woodman et al. (1993), this will be an interactive process. Hiring the best people with relevant job skills, reasonable social skills, and some evidence of capacity for creative thinking would of course be a first step. Using some type of survey to tap both social and creativity inclinations may be helpful (e.g., Runco et al., 2001; Scratchley and Hakstian, 2001). Next, one would want to educate employees about the basics of effective group and meeting sessions and then actually implement some of the best practices at times when breakthrough ideas are needed. Third, for those involved in team activities that include innovation, an emphasis on the importance of broad-based communication within the teams and with other teams can ensure that there is a full tapping of the collective intelligence or diverse knowledge base of the organizations (Hülsheger et al., 2009). Others have used the term *knowledge integration* (Sheremata, 2000) to refer to this process and claim that it is an important antecedent to fostering team innovation (Gebert et al., 2010). Exchange of information at a team level expands the knowledge and information (cognitive resources; Amabile and Khaire, 2008) available to the team, promotes enhanced analyses of the problems, and better assessment of the potential solutions to the problems (Nemeth and Owens, 1996). Exchange of ideas at a team level also enhances the diversity of ideas, creates new information through external networks (Perry-Smith, 2006), produces unexpected ideas (Simonton, 1991) and cross-fertilization of ideas (Ancona and Caldwell, 1992). Sharing and exchange of information helps in building on each other’s ideas (cognitive stimulation) resulting in additional and often unique associations between ideas. In terms of idea implementation, a team-based exchange of information promotes applications of solutions that suit the needs of all parties concerned (Drach-Zahavy and Somech, 2002). Team members’ socialization inside and outside of the workplace promotes openness to express ideas and opinions, which enhances a team’s engagement in creative tasks (Gilson and Shalley, 2004). However, it is also important to provide team members time for reflection related to the creative processes (Fay et al., 2015).

The development of effective creative processes in teams will require some team training and a culture that emphasizes psychological safety and a support for risk-taking. Furthermore, there needs to be a follow-up process in which the team members are apprised of what is being done with the proposed ideas further up the organization structure. To keep team members motivated for creative activity, there has to be some evidence of an impact of their efforts. Of course, there is a limit to the number of radical ideas that can be implemented or developed. However, if team members are collectively involved in the subsequent selection and development processes, this should allow them to experience the benefit of their efforts and develop a sense of collective creative efficacy (Taggar, in press). Periodic meetings of top management with rank and file team members about the innovative process and how to improve it should also be helpful and would provide a positive signal to employees that their personal perspectives and creativity are valued.

By utilizing the talents of creative people through effective group, meeting, and team processes and by developing an organizational culture and management team that supports innovation and has the leadership skills to manage the different phases of the innovation process effectively, organizations may discover their real innovative potential. However, to gain compelling evidence for this approach, it would be useful to have some intervention studies to determine the impact of implementing the best group creativity practices we have suggested. Thus far we know of no attempts to do these types of interventions in organizations (cf. Anderson et al., 2014).

## Research Gaps and Future Developments

In this paper we have tried to partially fill the gap that exists between experimental research on collaborative idea generation and its potential application for enhancing innovation in organizations. However, there are still many gaps in our knowledge about the most effective ways to use collaborative ideation in organizations. We have noted a number of them in our prior discussions. Although we have conducted two studies in organizations that have replicated our experimental findings (Paulus et al., 1995, 2015), there is a lack of research on actual collaborative ideation in organizations and its impact on organizational innovation. There are many factors in organizations that make such research difficult to conduct. However, we hope that in the future some open minded organizations will accommodate this type of research. It is particularly important to gain a better understanding of the evaluation and selection processes that follow idea generation sessions and how to insure that the best ideas “rise to the top.” Thus far there is very limited research on the connection between the divergent and convergent stages of collaborative ideation.

Although we have suggested best practices based on the research literature, there is a need for additional studies to compare various approaches to determine which ones are most optimal. How will self-selection of individuals to groups influence the creative process? This may allow for similar personalities to converge to the same groups. For example, this might make a group of introverts more comfortable in openly expressing ideas. How long should ideation sessions be? How should breaks be scheduled and what should happen during these breaks? What are ways to enhance the effectiveness of the writing and electronic methods? How does group size affect the outcomes of collaborative writing sessions? What is the optimal technique for collaborative writing? Is there some ideal combination of oral and written approaches to collaborative ideation? What is the best way to balance and integrate individual and group ideation sessions? How can some of the insights from this literature be used to enhance problem solving meetings? Future developments in computer science may enable the development of feedback systems that facilitate the innovation process. Such systems might provide feedback on the quality (e.g., novelty) of the ideas being generated in an electronic format and enable groups to focus their attention on those ideas for building and development. We have considerable knowledge about factors that influence individual creativity, and some of the groups literature suggests that these characteristics may also be influential in interactive settings. However, we know very little about the role of group level diversity in these characteristics in different phases of the innovation process. Will certain combinations of personalities and abilities in a team enable it to excel in the different phases?

We have come a long way in understanding the collaborative creativity process, but there is still much to learn about the optimal conditions, processes, and group compositions. Hopefully, future research in organizational contexts will fill some of these gaps.

## Author Contributions

PP wrote the first draft. JB did the next draft. JK contributed to a third draft.

## Conflict of Interest Statement

The authors declare that the research was conducted in the absence of any commercial or financial relationships that could be construed as a potential conflict of interest.
